# Supplementary figures and images for: The mesoscale organization of syntaxin 1A and SNAP25 is determined by SNARE–SNARE interactions
Source: eLife. 2021 Nov 15;10:e69236. doi: 10.7554/eLife.69236 (PMC8629428; doi:10.7554/eLife.69236)

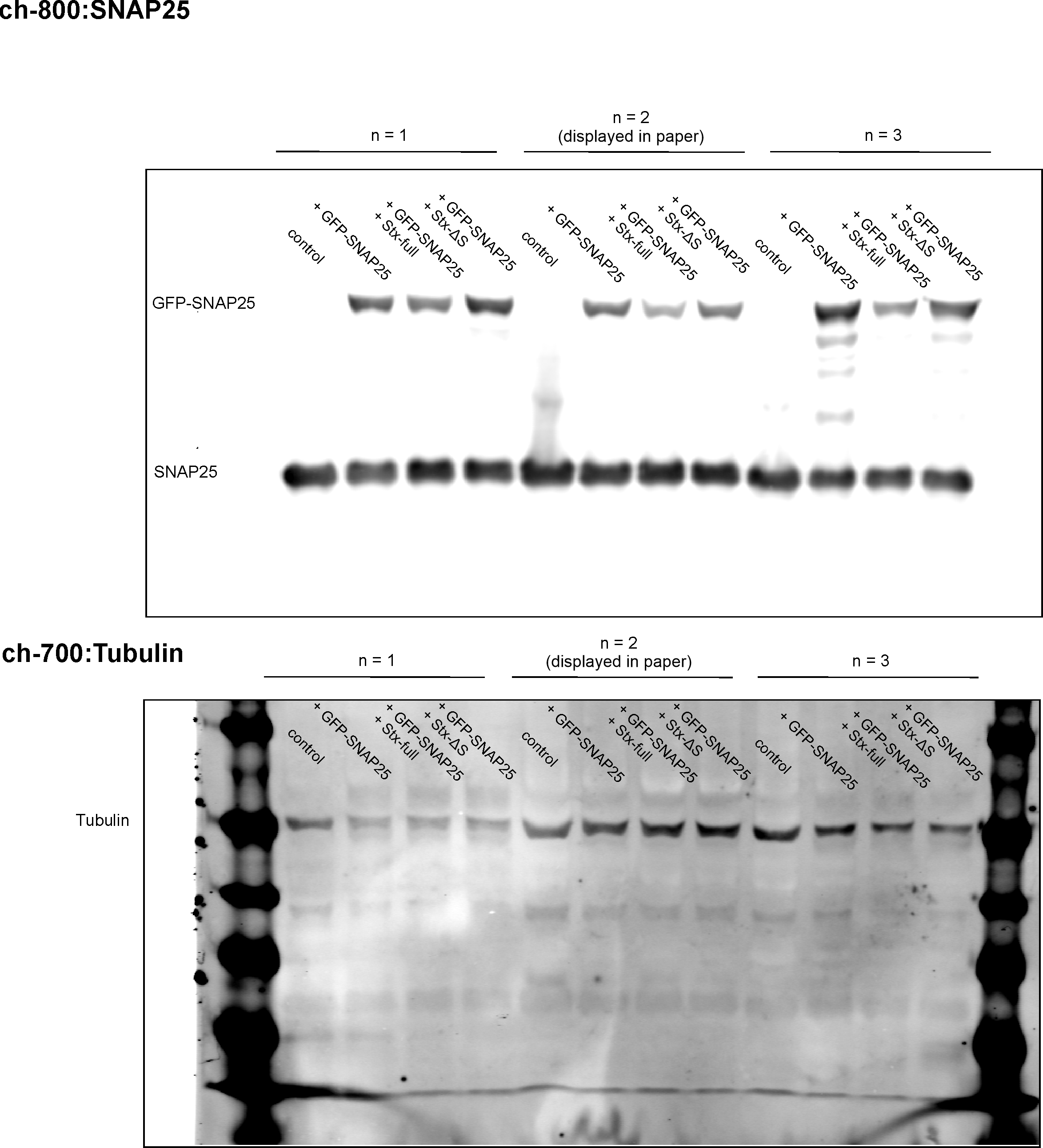

Supplement: Figure 1—source data 1. [file elife-69236-fig1-data1.zip › Fig.01-source data 01/fullBlot_SNAP25.png]

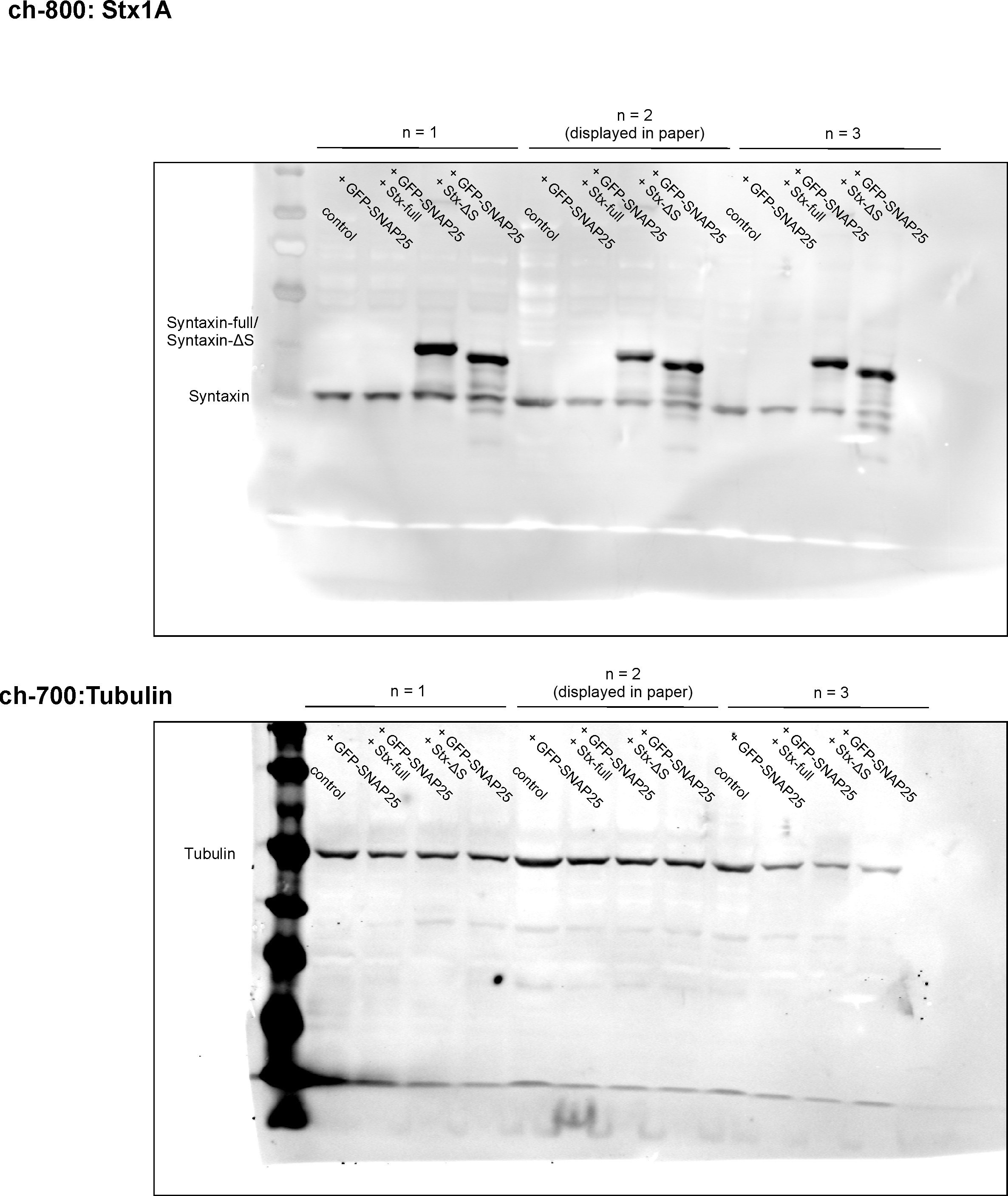

Supplement: Figure 1—source data 1. [file elife-69236-fig1-data1.zip › Fig.01-source data 01/fullBlot_Stx.png]

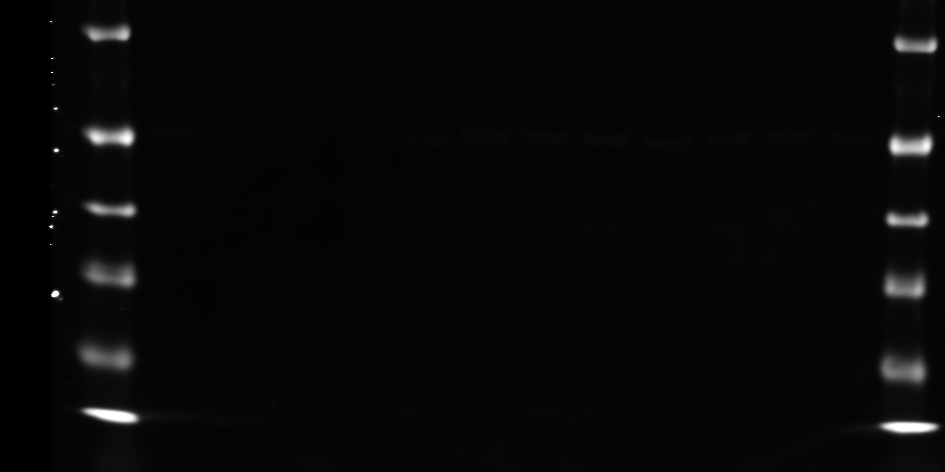

Supplement: Figure 1—source data 1. [file elife-69236-fig1-data1.zip › Fig.01-source data 01/SNAP25-raw images/ch-700_Tubulin_raw.TIF]

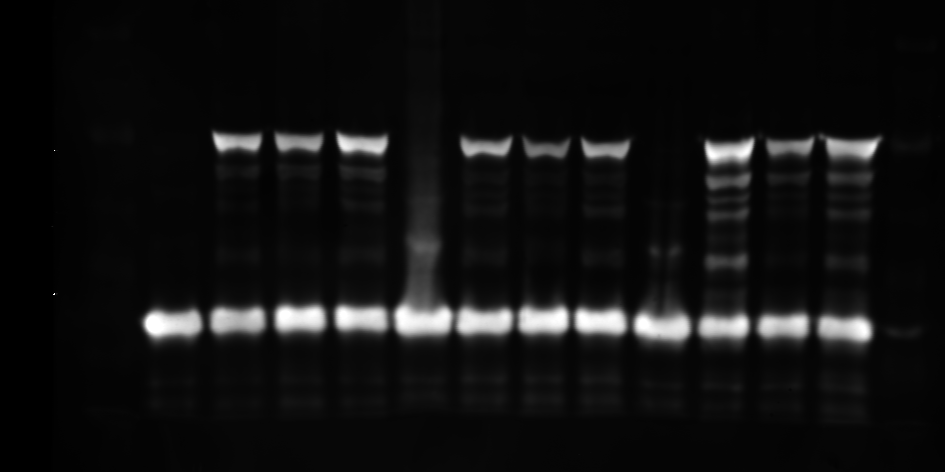

Supplement: Figure 1—source data 1. [file elife-69236-fig1-data1.zip › Fig.01-source data 01/SNAP25-raw images/ch-800_SNAP25_raw.TIF]

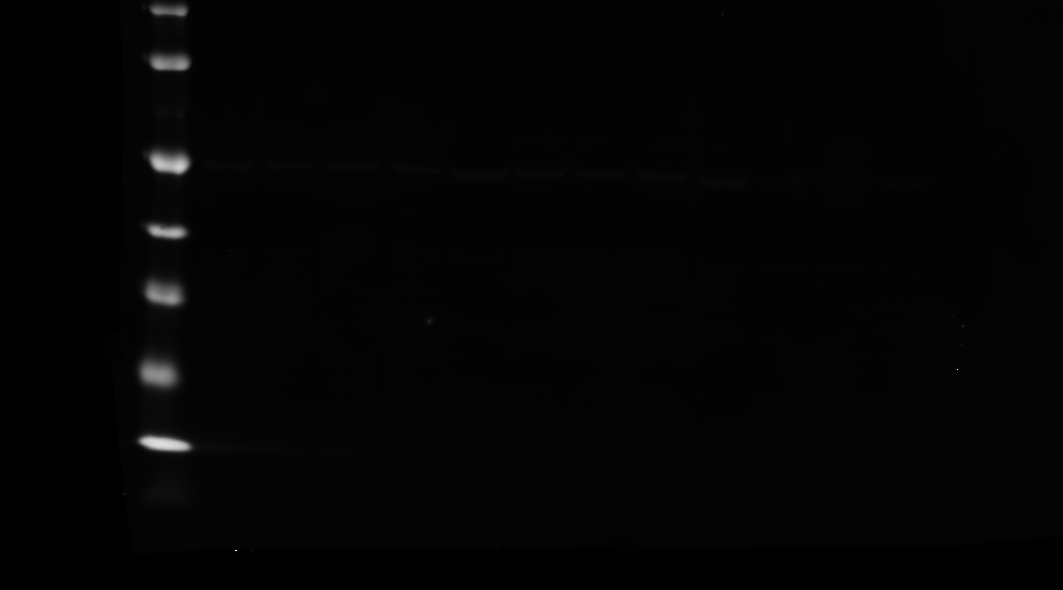

Supplement: Figure 1—source data 1. [file elife-69236-fig1-data1.zip › Fig.01-source data 01/Stx1A-raw images/ch-700_Tubulin_raw.TIF]

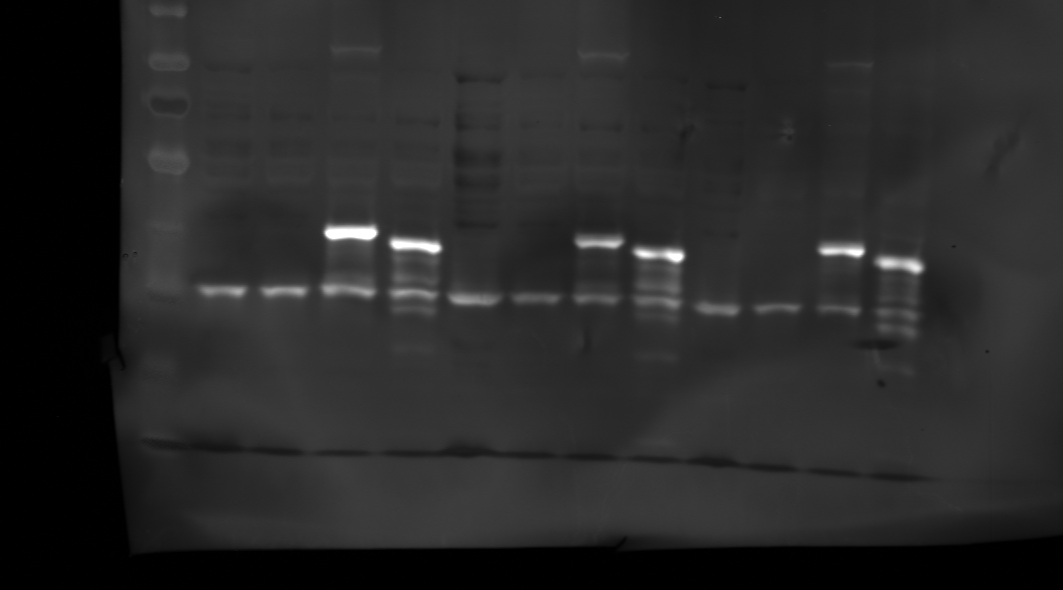

Supplement: Figure 1—source data 1. [file elife-69236-fig1-data1.zip › Fig.01-source data 01/Stx1A-raw images/ch-800_HPC-1_raw.TIF]

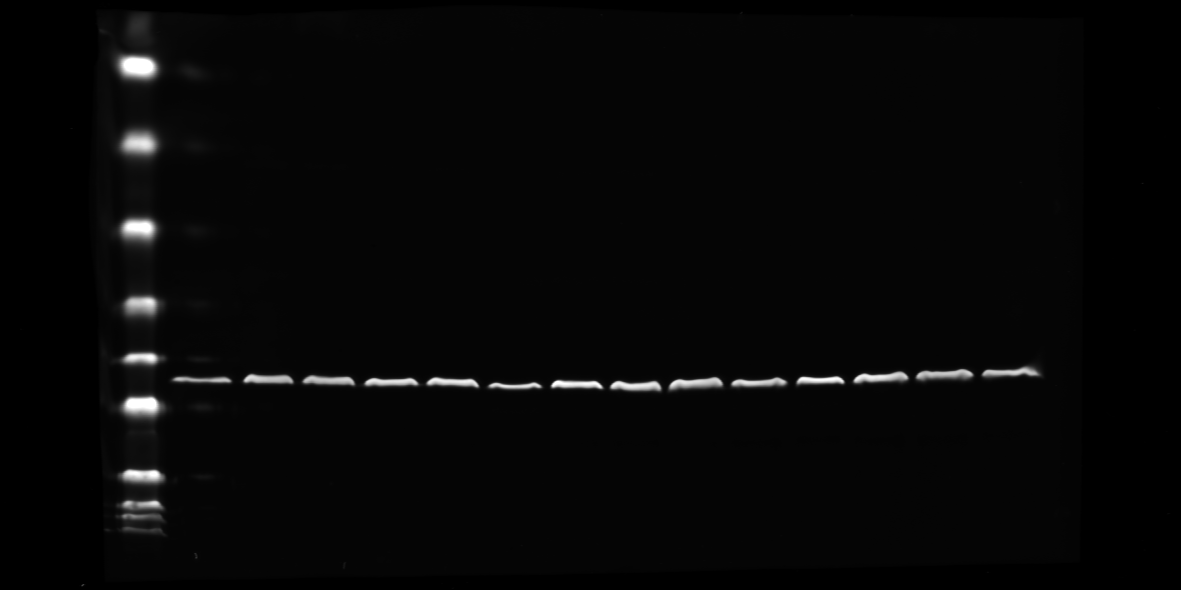

Supplement: Figure 1—figure supplement 1—source data 1. [file elife-69236-fig1-figsupp1-data1.zip › Fig.01-suppl.01-source data 01/ch700_actin_raw-image.TIF]

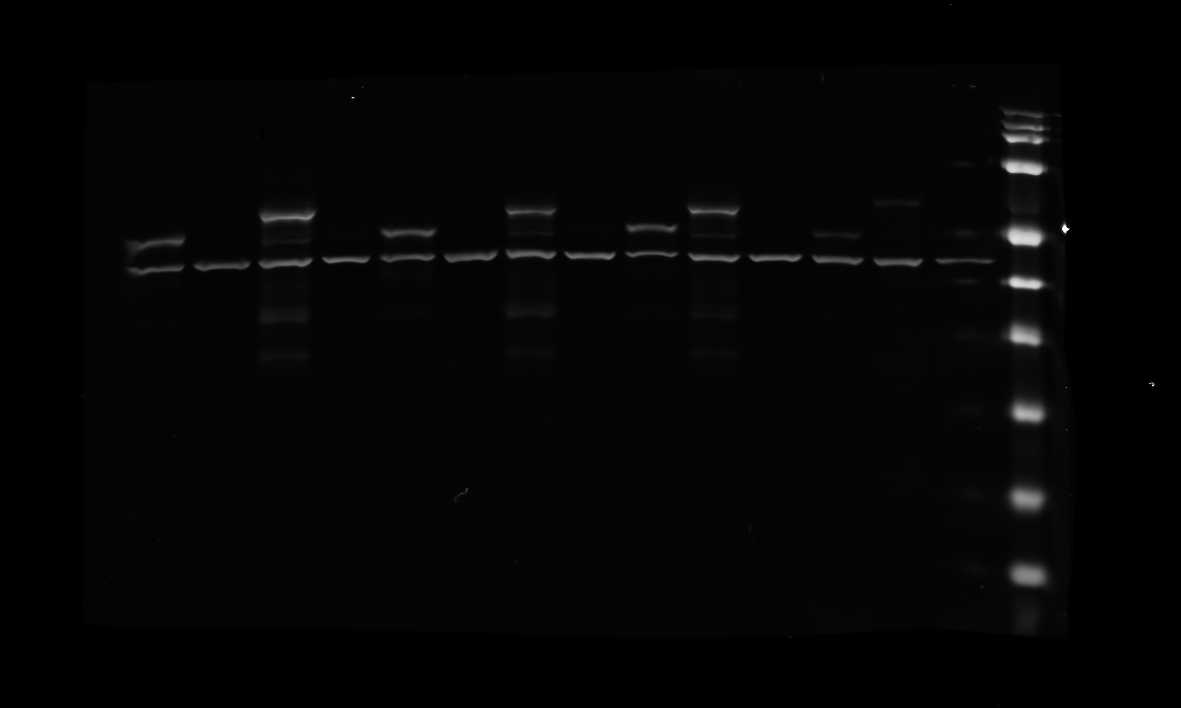

Supplement: Figure 1—figure supplement 1—source data 1. [file elife-69236-fig1-figsupp1-data1.zip › Fig.01-suppl.01-source data 01/ch700_GFP_raw-image.TIF]

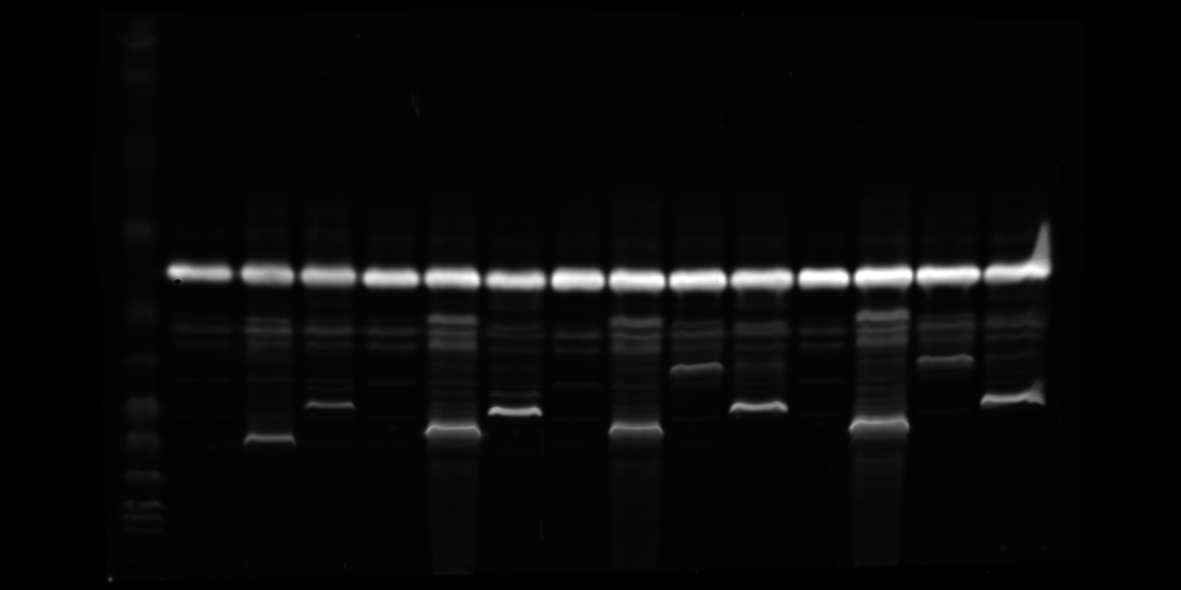

Supplement: Figure 1—figure supplement 1—source data 1. [file elife-69236-fig1-figsupp1-data1.zip › Fig.01-suppl.01-source data 01/ch800_stx-snap_raw-image.TIF]

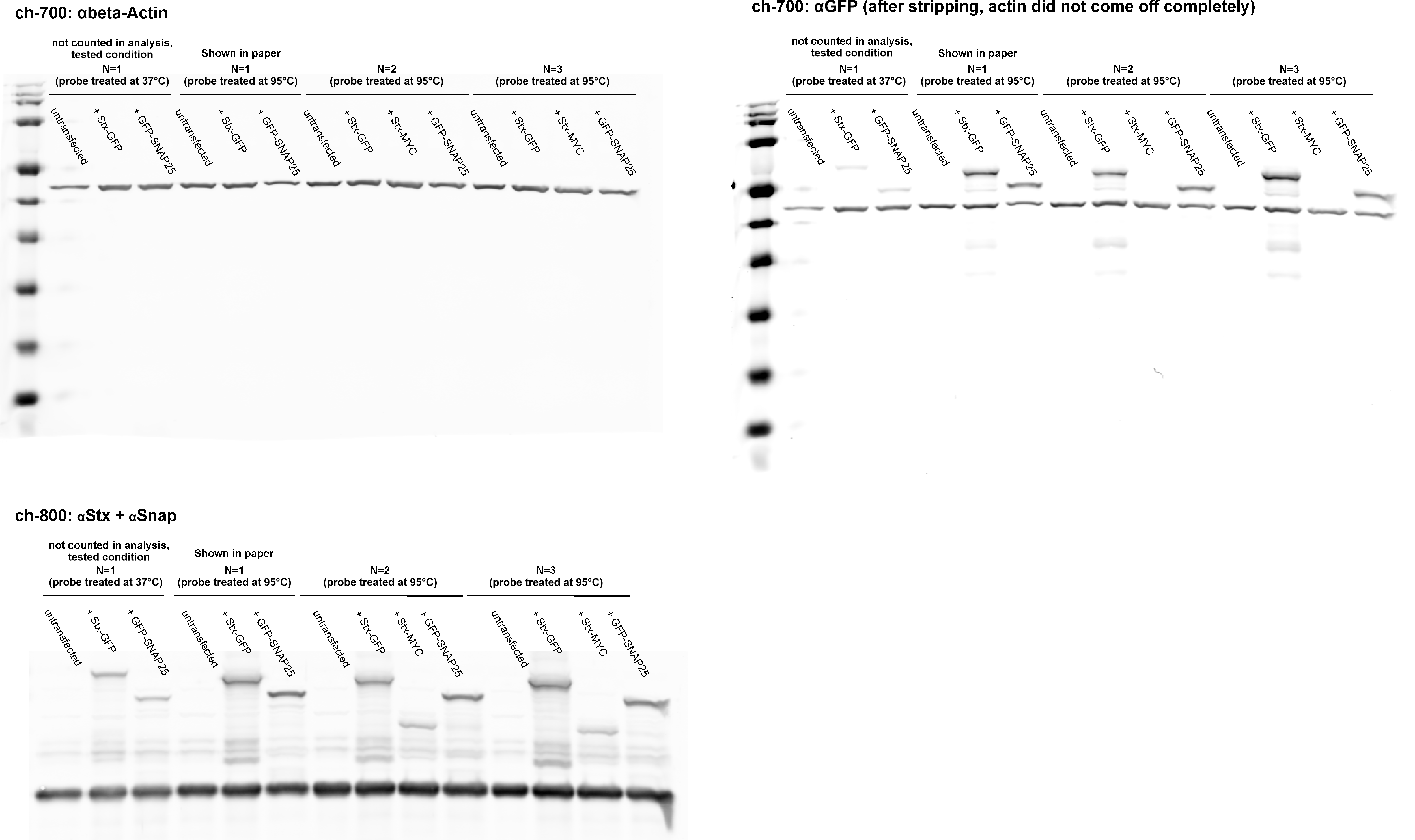

Supplement: Figure 1—figure supplement 1—source data 1. [file elife-69236-fig1-figsupp1-data1.zip › Fig.01-suppl.01-source data 01/GFP-ratio_fullBlot.png]

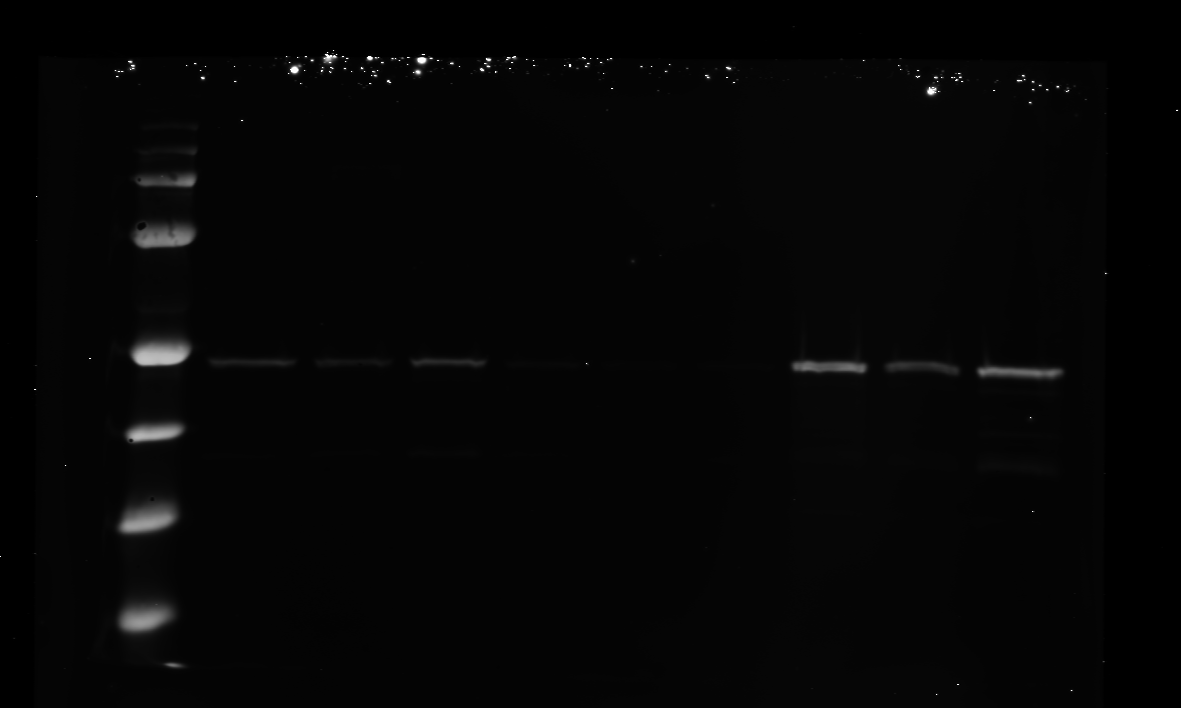

Supplement: Figure 3—source data 1. [file elife-69236-fig3-data1.zip › Fig.03-source data 01/ch-700_GFP_raw-image.TIF]

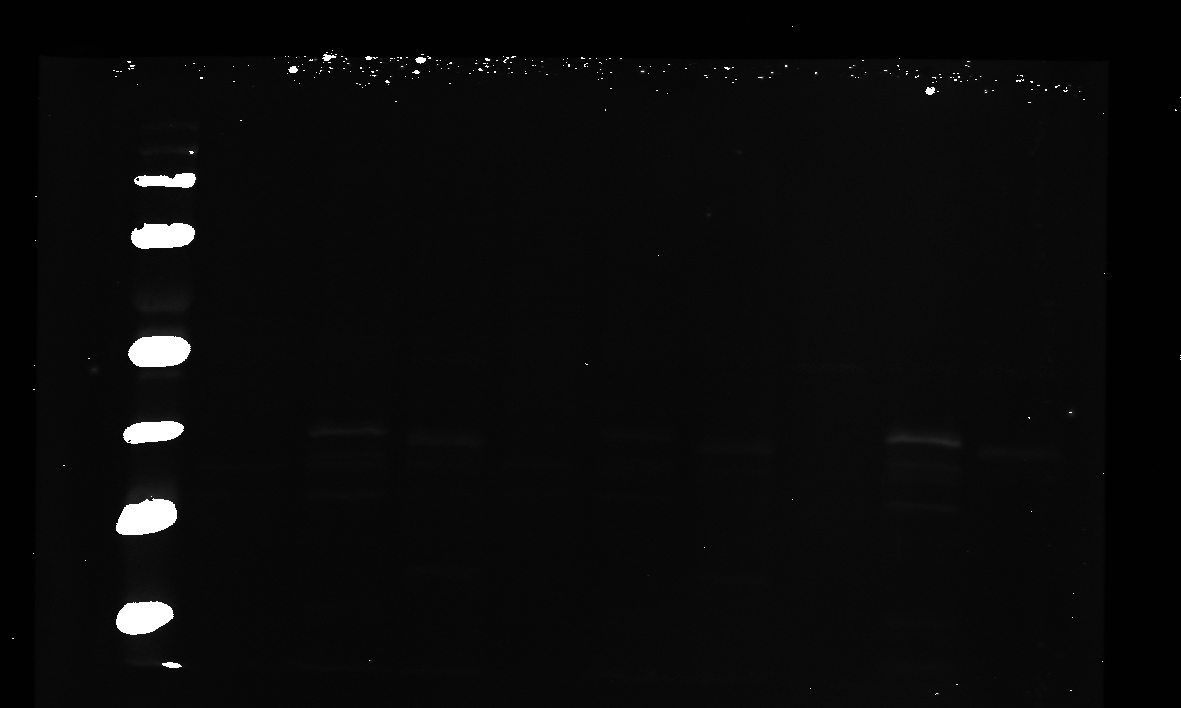

Supplement: Figure 3—source data 1. [file elife-69236-fig3-data1.zip › Fig.03-source data 01/ch-800_Myc_raw-image.TIF]

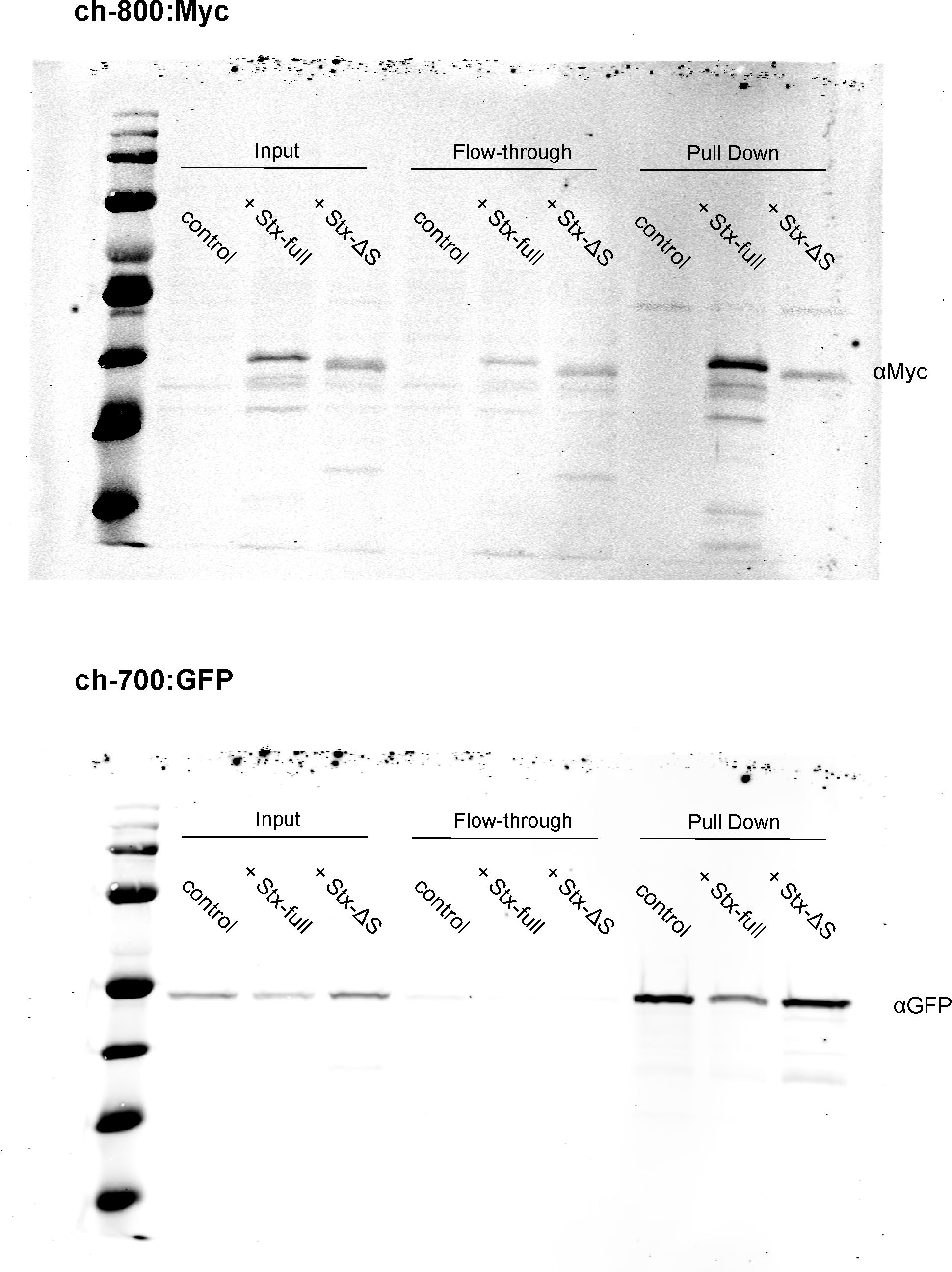

Supplement: Figure 3—source data 1. [file elife-69236-fig3-data1.zip › Fig.03-source data 01/Co-IP_fullBlot.png]
